# Taxono-genomics description of *Olsenella lakotia *SW165 ^T^ sp. nov., a new anaerobic bacterium isolated from cecum of feral chicken

**DOI:** 10.12688/f1000research.25823.1

**Published:** 2020-09-08

**Authors:** Supapit Wongkuna, Sudeep Ghimire, Tavan Janvilisri, Kinchel Doerner, Surang Chankhamhaengdecha, Joy Scaria

**Affiliations:** 1 Doctor of Philosophy Program in Biochemistry (International Program), Department of Biochemistry, Faculty of Science, Mahidol University, Bangkok, 10400, Thailand; 2 Department of Veterinary and Biomedical Sciences, South Dakota State University, Brookings, South Dakota, 57007, USA; 3 South Dakota Center for Biologics Research and Commercialization, Brookings, South Dakota, 57007, USA; 4 Department of Biochemistry, Faculty of Science, Mahidol University, Bangkok, 10400, Thailand; 5 Department of Biology and Microbiology, South Dakota State University, Brookings, South Dakota, 57007, USA; 6 Department of Biology, Faculty of Science, Mahidol University, Bangkok, 10400, Thailand

**Keywords:** Olsenella lakotia SW165T sp. nov., culturomics, chicken gut microbiota, taxono-genomics

## Abstract

**Background: **The microbial community residing in the animal gastrointestinal tract play a crucial role in host health. Because of the high complexity of gut microbes, many microbes remain unclassified. Deciphering the role of each bacteria in health and diseases is only possible after its culture, identification, and characterization. During the culturomics study of feral chicken cecal sample, we cultured a possible novel strain SW165 ^T^.

**Methods: **For the possible novel strain SW165 ^T^, phenotypic characterization was performed using colony morphology, Gram staining, growth in different temperature and pH and motility. Biochemical assays included carbon source utilization, enzymatic activity, cellular fatty acids and short chain fatty acid production. 16S rRNA sequencing and whole genome sequencing and comparison was performed for genetic analysis.

**Results: **This strain was isolated from cecal content of feral chickens in Brookings, South Dakota, USA. Phylogenetic analyses based on 16S rRNA gene sequence revealed that the closest valid neighbor was *Olsenella profusa* DSM 13989 ^T ^(96.33% similarity) within the family *Atopobiaceae*. Cells were Gram-strain-positive and obligately anaerobic bacilli in chains. The optimum temperature and pH for the growth of the microorganism were 37-45 ^o^C and pH 6.0-7.5 respectively.  This strain produced acetic acid as the primary fermentation product. Major fatty acids were C _12:0_, C _14:0_, C _14:0_ DMA and summed feature 1 (C _13:1_ at 12-13 and C _14:0_ aldehyde). Strain SW165 ^T^ exhibited a genome size of 2.43 Mbp with a G+C content of 67.59 mol%, which is the second highest G+C content among members of the genus *Olsenella*. The digital DNA-DNA hybridization and OrthoANI values between SW165 ^T^ and DSM 13989 ^T^ were only 17.6 ± 5.3 and 74.35%, respectively.

**Conclusion:** Based on the phenotypic, biochemical, and genomic analyses, we propose the new species of the genus *Olsenella, *and name it *Olsenella lakotia* SW165 ^T^ sp. nov., (=DSM 107283 =CCOS 1887) as the type strain.

## Introduction

The chicken gut harbors highly diverse microbes ^1^. The gut microbes are known for their nutritional benefits by producing short chain fatty acids, enzymes, amino acids along with their ability to resist pathogens, immunity development and maintain homeostasis ^2^. Even though culture independent methods have highlighted the functional capability of gut microbes, validation of these functions requires their cultivation, identification, and characterization. Most of the intestinal bacteria have been never isolated in the laboratory ^3, 4^ thus hindering the understanding of their ecological and functional roles in the gut. Recently, “culturomics” strategy drives discovery of previously uncultured species based on modified culture conditions, such as media, temperature, pH and atmosphere and rapid identifying methods; matrix-assisted desorption ionization- time of flight mass spectrometry (MALDI-TOF MS) and 16S rRNA gene sequencing ^5– 7^. We employed culturomics to isolate bacteria from cecum of feral chickens. Based on bacterial isolation and identification, strain SW165 ^T^ was found as a new species within the genus *Olsenella*.

The members of the genus *Olsenella* are strictly anaerobic, Gram-positive, non-motile, non-spore-forming bacilli or cocci. This genus was first named by Dewhirst *et al.* 2001 ^8^ and amended by Zhi et al 2009 ^9^ and Kraatz et al 2011 ^10^. This genus has recently been reclassified as a member of the family *Atopobiaceae* under order Coriobacteriales, class Coriobacteria, and phylum Actinobacteria ^11^. The genus *Olsenella* consists of nine published species; *O. uli* ^12^, *O. profusa* ^8^, *O. umbonata* ^10^, *O. scatoligenes* ^13^, *O. urininfantis* ^14^, *O. congonensis* ^15^, *O. provencensis* ^16^, *O. phocaeensis* ^16^, and *O. mediterranea* ^16^. The members of this genus are strictly anaerobic, Gram positive, non-motile, non-spore forming rod shaped with G+C content of DNA 62–64% ^8, 13, 17^. The main habitats of the *Olsenella* are the oral cavity and gastrointestinal tract of humans ^18– 21^, animals and various anaerobic environmental sites ^22– 24^. In chicken cecum, many members of genus *Olsenella* have been reported in the chicken microbiome in metagenomic-based studies ^15, 25^. However, only *O. uli* was isolated from chicken gut ^26^.

The taxono-genomics approach uses combination of phenotypic and genotypic characterization to describe new bacteria ^27, 28^. Phenotypic investigation includes morphological, physiological, and biochemical assays. Genome-based and 16S-based analysis are used in genotypic characterization. In this study, strain SW165 ^T^ was described using taxono-genomics and compared to its closely related phylogenetic neighbors. Following analysis, we found that strain SW165 ^T^ belongs to a novel species for which the name *Olsenella lakotia* SW165 ^T^ sp. nov. is proposed.

## Methods

### Strain isolation

The cecal content of feral chickens was collected in Brookings, South Dakota, USA. For cultivation, the samples were transferred into an anaerobic workstation (Coy Laboratory) containing 85% nitrogen, 10% hydrogen and 5 % carbon dioxide and plated on a modified Brain Heart Infusion (BHI-M) agar containing 37 g/L of BHI, 5 g/L of yeast extract, 1 ml of 1 mg/ml menadione, 0.3 g of L-cysteine, 1 ml of 0.25 mg/L of resazurin, 1 ml of 0.5 mg/ml hemin, 10 ml of vitamin and mineral mixture, 1.7 ml of 30 mM acetic acid, 2 ml of 8 mM propionic acid, 2 ml of 4 mM butyric acid, 100 µl of 1 mM isovaleric acid, and 1% of pectin and inulin. After 3 days of incubation at 37°C under anaerobic conditions, single colony of strain SW165 was identified by MALDI-TOF mass spectrometry using a Microflex spectrometer (Bruker Daltonics, Bremen, Germany). The strain was maintained in BHI-M medium and stored with 10% (v/v) Dimethyl Sulfoxide (DMSO) at -80°C.

### Phenotypic and biochemical tests

For morphological characterization, the strain SW165 ^T^ was anaerobically cultivated in BHI-M medium, pH 6.8-7.2, at 37 ^o ^C. Colony morphologies were examined after 2–3 days of incubation on BHI-M agar plates. Gram-staining was performed using a Gram-Staining kit set (Difco), according to the manufacturer’s instructions. Cell morphologies were examined by scanning electron microscopy (SEM) of cultures during exponential growth. Aerotolerance was examined by incubating cultures for 2 days separately under aerobic and anaerobic conditions. Growth of strain SW165 ^T^ at 4, 20, 30, 37, 40 and 55°C was determined. For determining the range of pH for growth of SW165 ^T^, the pH of the medium was adjusted to pH 4.0–9.0 with sterile anaerobic stock solutions of 0.1 M HCl and 0.1 M NaOH. Motility of this microorganism was determined using motility medium with triphenyltetrazolium chloride (TTC) ^29^. The growth was indicated by the presence of red color, reduced form of TTC after it is absorbed into bacterial cell wall. 

Biochemical tests to determine standard taxonomic characteristics for strain SW165 ^T ^were performed in triplicate. The utilization of various substrates as sole carbon and energy sources and enzyme activities were performed using the AN MicroPlate (BIOLOG) and API ZYM (bioMérieux) according to the manufacturer's instructions. Reference strain, DSM 13989 ^T ^purchased from the DSMZ culture collection and isolated strains SW165 ^T^ were simultaneously cultured in BHI-M medium at 37°C for 24 h under anaerobic condition before cell biomass were harvested for cellular fatty acid analysis. The fatty acids were extracted, purified, methylated and analyzed using gas chromatography (Agilent 7890A) based on the instruction from Microbial Identification System (MIDI) ^30^. Metabolic end-products such as short-chain fatty acids of strain SW165 ^T^ and DSM 13989 ^T^ grown in BHI-M were determined using a gas chromatography. The cultures were deproteinized with 25% metaphosphoric acid before supernatant collection. The supernatant was analyzed for the presence of acetic acid, butyric acid, isovaleric acid and propionic acid using GC (Themo Scientific ^TM^ TRACE ^TM^ 1310 GC equipped with a TraceGOLD ^TM^ TG-WaxMS A GC column.). 

### 16S RNA phylogenetical analysis

Genomic DNA of the strain SW165 ^T^ was extracted using a DNeasy Blood & Tissue kit (Qiagen), according to the manufacturer’s instructions. 16S rRNA gene sequence was amplified using universal primer set 27F (5’- AGAGTTTGATCMTGGCTCAG-3’; Lane *et al.,* 1991) and 1492R (5’- ACCTTGTTACGACTT- 3’; Stackebrandt *et al.,* 1993) ^31, 32^, and sequenced using a Sanger DNA sequencer (ABI 3730XL; Applied Biosystems). The 16S rRNA gene sequence of SW165 was then compared with closely related strains from the GenBank ( www.ncbi.nlm.nih.gov/genbank/) and EZBioCloud databases ( www.ezbiocloud.net/eztaxon) ^33^. Alignment and phylogenetic analysis were conducted using MEGA7 software ^34^. Multiple sequence alignments were generated using the CLUSTAL-W ^35^. Reconstruction of phylogenetic trees was carried out using the maximum-likelihood (ML) ^36^, maximum-parsimony (MP) ^37^, and neighbor-joining (NJ) ^38^ methods. The distance matrices were generated according to Kimura's two-parameter model. Bootstrap resampling analysis of 1000 replicates was performed to estimate the confidence of tree topologies. 

### Genome sequencing and analysis

The whole genome sequencing of strain SW165 ^T^ was performed using Illumina MiSeq sequencer using 2x 300 paired end V3 chemistry. The reads were assembled using Unicycler that builds an initial assembly graph from short reads using the de novo assembler SPAdes 3.11.1 ^39^. The quality assessment for the assemblies was performed using QUAST5.0.2 ^40^. Genome annotation was performed using Rapid Annotation using Subsystem Technology (RAST) server ^41^. The digital DNA-DNA hybridization (dDDH) was performed using Genome-to-Genome Distance Calculator (GGDC) web server ( http://ggdc.dsmz.de) to estimate the genomic similarity between strain SW165 ^T^ and the closest phylogenetic neighbor. Average nucleotide identity (ANI) between strain SW165 ^T^ and the closely related strains was also calculated using the OrthoANI software ^42^. Distribution of functional categories of strain SW165 ^T^ was compared to *Olsenella* species and was presented in a heatmap generated using Explicet version 2.10.5 ^43^.

## Results

**Figure 1.**
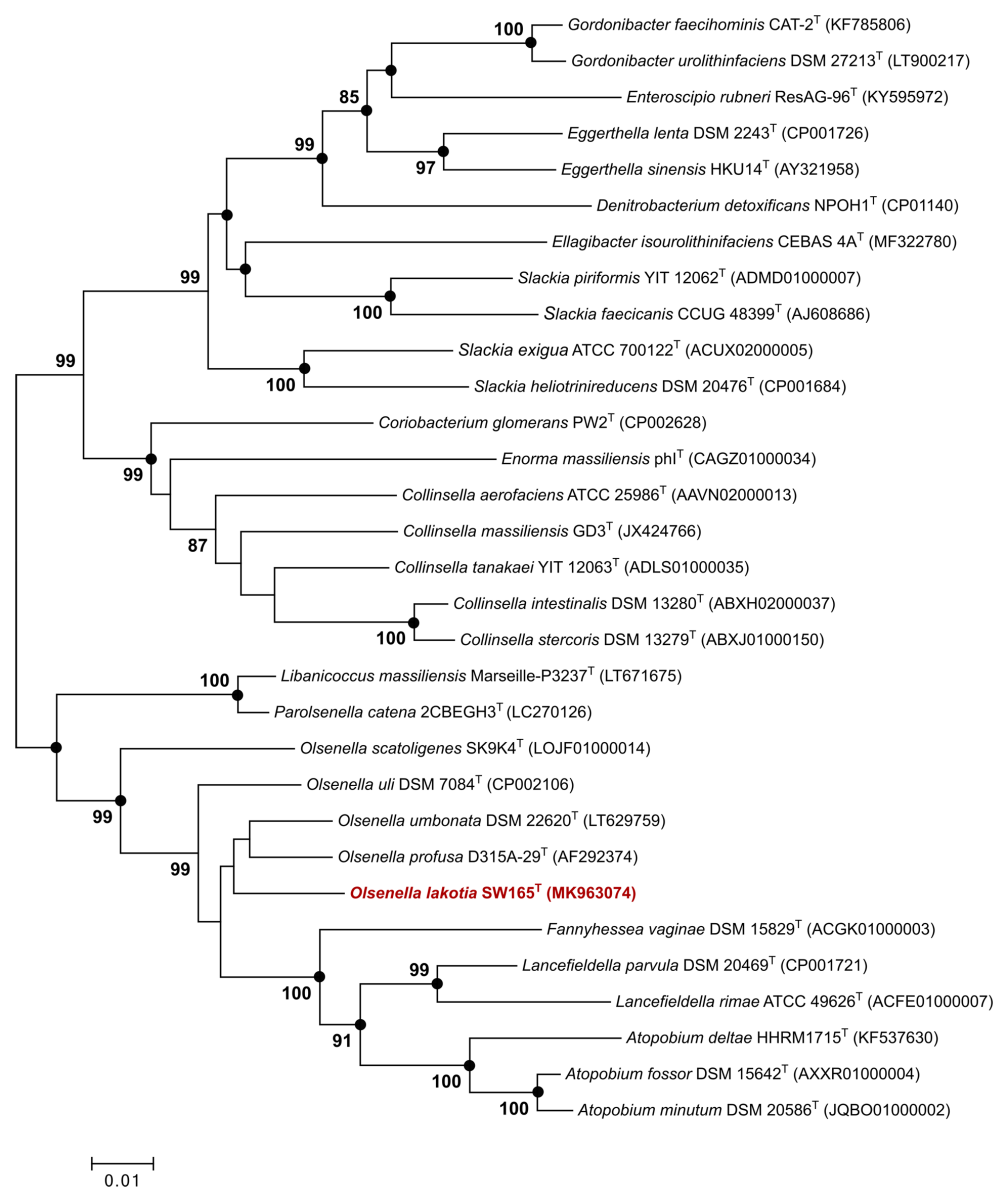
16S rRNA based neighbor-joining tree of SW165 ^T^ and its neighbors. Tree shows phylogenetic position of *Olsenella lakotia* DSM 107283 ^T^ and closely related species in the family *Atopobiaceae*. GenBank accession numbers of the 16S rRNA gene sequences are given in parentheses. Black circles indicate that the corresponding branches were also recovered both by maximum-likelihood and maximum parsimony methods. Bootstrap values (based on 1000 replications) greater than or equal to 70% are shown in percentages at each node. Bar, 0.01 substitutions per nucleotide position.

Strain SW165 ^T^ was isolated from cecal contents of feral chicken in anaerobic chamber (Coy Laboratory Product, MI, USA). Colonies of SW165 ^T^ on BHI-M agar were 0.2–0.5 cm in diameter, appeared white, smooth, and umbonate with entire circular edges when grown at 37°C anaerobically after 48 hours of incubation. After cultivation, the colonies of this strain were subjected to identification by MALDI-TOF using a Microflex spectrometry (Bruker Daltonics, Bremen, Germany). MALDI-TOF did not identify the strain as the scores obtained were < 1.70. Thus, full length 16S rRNA gene was sequenced using Sanger sequencing method. The 16S rRNA of the strain SW165 ^T^ showed 96.33% identity with *O. profusa* DSM 13989 ^T ^(GenBank accession no. AF292374), the validly closest species within phylogenetical nomenclature ( Figure 1). The current cut off for species delineation from its nearest neighbor based on 16S rRNA is 98.7% ^44^. As the identity of 16S rRNA of strain SW165 ^T^ was lower than threshold, it was considered as a representative of putatively novel species within the genus *Olsenella* in the family *Atopobiaceae.* Phylogenetically, the strain was found to cluster together with other members of genus *Olsenella,* as shown in Figure 1, validating that SW165 ^T^ belongs to genus *Olsenella* taxonomically. 

**Figure 2.**
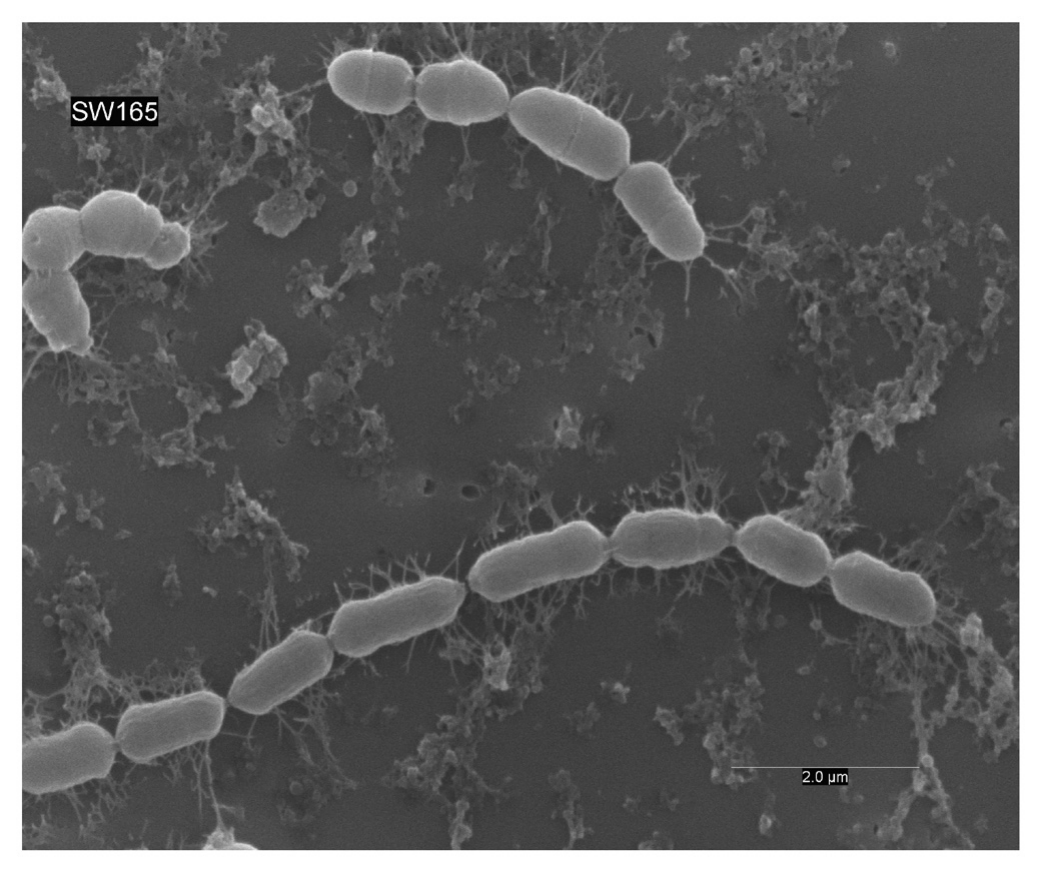
Scanning electron micrograph of *O. lakotia* SW165 ^T^. Cells were anaerobically cultured for 24 hours at 37 ^o ^C in BHI-M medium. Bar, 2 μm; uncropped/unedited image.

Phenotypic growth of strain SW165 ^T^ was observed on modified BHI-M agar after 2–3 days of incubation at temperature between 37°C and 45°C and pH between 6.0–7.0. The optimum temperature and pH for the growth were at 45°C and pH 7.0, respectively. Strain SW165 ^T^ grew only under anaerobic conditions, suggesting obligate anaerobic nature. Bacterial cells were Gram-stain-positive bacilli (0.5–2.0 µm), growing in pairs or as short chains and were non-motile ( Figure 2).

To further analyze the biochemical properties of the strain, we performed the carbon source utilization assay using BIOLOG AN microplate and compared it to closely related taxa. Strain SW165 ^T^ consumed various carbon sources for the growth, which differed from related strains in the utilization of D-fructose, L-fucose, D-galactose, maltose, D-melibiose and D-raffinose, and in the non-utilization of dulcitol. Based on enzymatic activity test, the strain produced several enzymes, including alkaline phosphatase, leucine arylamidase, cysteine arylamidase, α-galactosidase, β-galactosidase, β-glucuronidase, α-glucosidase, and β-glucosidase. Interestingly, alkaline phosphatase α-galactosidase, β-galactosidase and β-glucuronidase are not reported from its closest neighbors ( Table 1). Furthermore, the dominant cellular fatty acids of the strain SW165 ^T^ were saturated, including C _12 : 0 _(25.5%) and C _14 : 0 _(22.83%). Moreover, other dominant fatty acids were C _14 : 0_ DMA (15.61%) and summed feature 1 [C _13:1 _and/or C _14:0 _aldehyde; 13.94%]. However, there were distinct quantities of some fatty acids between SW165 ^T^ and the relative strains ( Table 2). The major short chain fatty acids produced by SW165 when cultivated in BHI-M were acetic acid (3.74 mM) followed by propionic acid (0.53 mM).

**Table 1.** Characteristics of *O. lakotia* SW165 ^T^ and closely related strains. Column headers show Strains designated in the following numbers: 1 - SW165 ^T^; 2 - *O. profusa* DSM 13989 ^T^; 3 - *O. uli* DSM 7084 ^T^; 4 - *O. umbonata* DSM 22620 ^T^; 5 - *O. scatoligenes* DSM 28304 ^T^. Results for metabolic end products of SW165 are from this study with cells that were cultured for 3 days at 37°C in BHI-M. +, positive; -, negative; w, weak; ND, not determined.

Characteristic	1	2	3 †	4 †	5 ‡
Gram stain	+	+	+	+	+
Growth at 37o C	+	+	+	+	+
Motility	-	-	-	-	-
Carbon source (BIOLOG AN)					
Arbutin	+	+	ND	ND	ND
D-Cellobiose	+	+	-	-	+
Dextrin	+	+	ND	ND	ND
D-Fructose	+	-	ND	ND	ND
L-Fucose	+	-	ND	ND	ND
D-Galactose	+	-	ND	ND	ND
α-D-Glucose	+	+	ND	ND	ND
Dulcitol	-	+	ND	ND	ND
Maltose	+	-	-	+	ND
D-Mannose	+	+	-	+	ND
D-Melibiose	+	-	-	-	ND
D-Raffinose	+	-	ND	ND	ND
Salicin	+	+	-	-	+
Sucrose	+	+	-	+	ND
Turanose	+	+	ND	ND	ND
Enzyme activity (API ZYM)					
Alkaline phosphatase	+	-	+	-	ND
Esterase (C 4)	-	-	+	+	-
Leucine arylamidase	+	+	+	+	ND
Cystine arylamidase	+	+	-	-	ND
α-chymotrypsin	w	-	ND	ND	ND
α-galactosidase	+	-	ND	ND	ND
β-galactosidase	+	-	-	-	+
β-glucuronidase	+	-	ND	ND	ND
α-glucosidase	+	+	-	+	+
β-glucosidase	+	+	+	-	+
Short-chain fatty acid production	A	L, a, f	L, a, f	L, a, f	L, a, f
DNA G+C content (mol%)	67.59	64	64.7	64.9	62.1

†Data from Kraatz *et al.* (2011) ^10^ ‡Data from *et al.* (2015) ^13^ A/a, acetic acid; L, lactic acid; f, formic acid. Capital letters indicate major end products.

**Table 2.** Cellular fatty acid compositions of *O. lakotia* SW165 ^T^ and related neighbors. Strains: 1 - SW165 ^T^; 2 - *O. profusa* DSM 13989 ^T^; 3 - *O. uli* DSM 7084 ^T^; 4- *O. umbonata* DSM 22620 ^T^; 5- *O. scatoligenes* DSM 28304 ^T^. Values are percentages of total fatty acids detected. Fatty acids with contents of less than 1% in all strains are not shown; ND, Not detected.

Fatty acid composition	1	2	3 ‡	4 ‡	5 ‡
Straight chain					
C10 : 00	3.34	ND	ND	ND	ND
C12 : 00	25.5	ND	2.8	ND	ND
C14 : 00	22.83	9.94	1.3	31.6	25.9
C16 : 00	2.69	5.82	4.3	6.2	2.7
C16 : 0 aldehyde	0.92	3.48	ND	ND	ND
Demethylacetal (DMA)					
C12 : 0 DMA	8.44	ND	ND	ND	ND
C14 : 0 DMA	15.61	4.33	ND	ND	ND
C16 : 0 DMA	1.21	13.97	ND	ND	ND
C18 : 0 DMA	0.65	1.18	ND	ND	ND
Branched					
C14 : 0 iso	ND	22.34	ND	ND	ND
C13 : 0 anteiso	ND	1.79	ND	ND	ND
C15 : 0 anteiso	ND	14.48	ND	ND	ND
C15 : 0 anteiso DMA	ND	5.17	ND	ND	ND
Unsaturated					
C18 : 1 *ω*9 *c*	1.82	3.9	69.8	20.7	25.7
C18 : 2 *ω*6,9 *c*	0.89	2.03	ND	ND	ND
Summed Feature 1	13.94	2.15	ND	11.3	20.7
Summed Feature 13	ND	5.17	ND	ND	ND

‡Data from *et al.* (2015) ^13^ ^*^Summed features are fatty acids that could not be separated using the MIDI System. Summed feature 1 contains C _13 : 1 _and/or C _14 : 0 _aldehyde. Summed feature 13 contains C _15 : 0 _anteiso and/or C _14 : 0 _2-OH.

**Figure 3.**
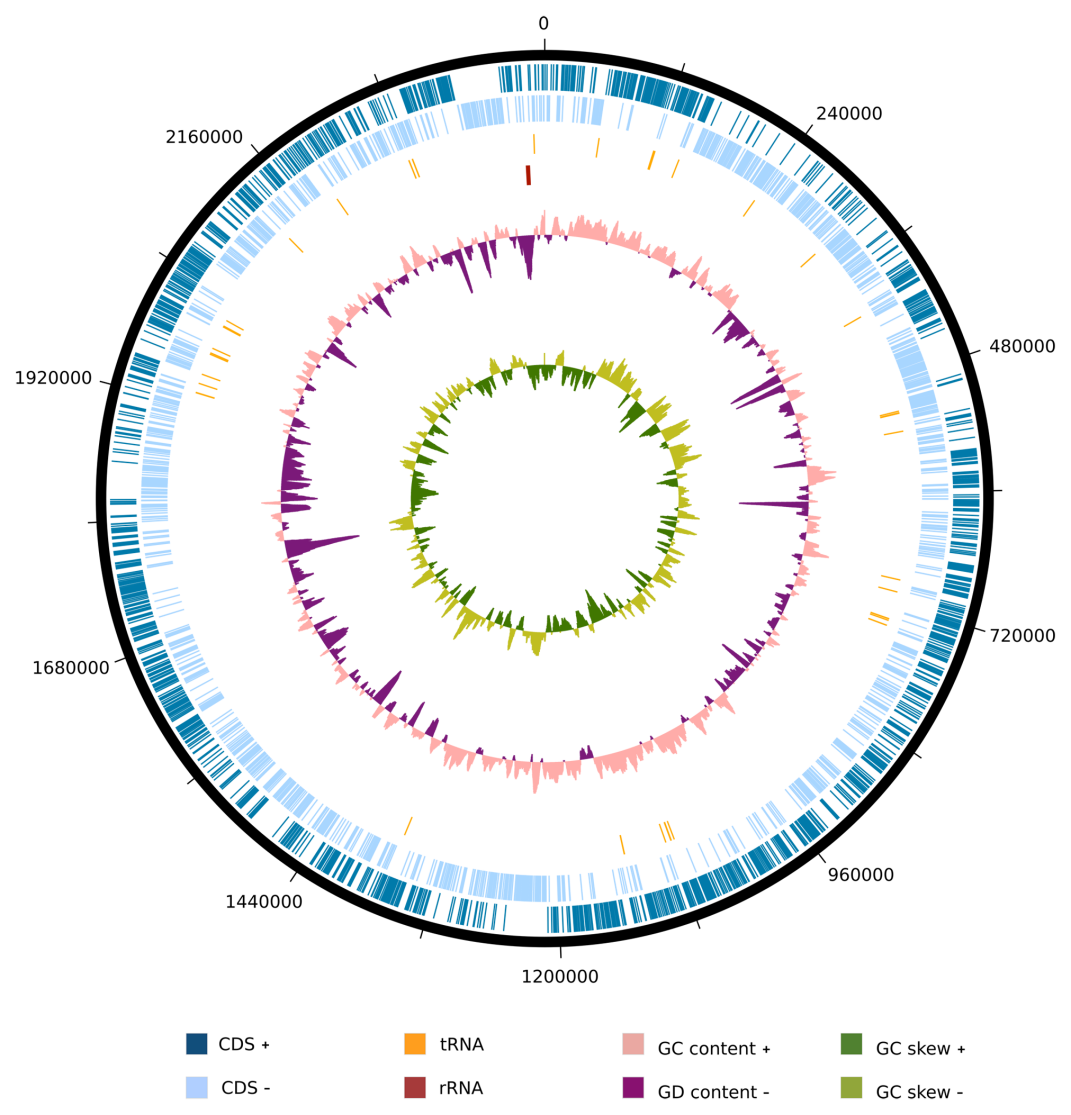
Graphical circular map of *O. lakotia* SW165 ^T^ genome. From outside to the center: coding sequences on the forward strand (CDS +), coding sequences on the reverse strand (CDS -), tRNAs, rRNAs, GC content, and GD skew.

We examined the genome of the strain SW165 ^T^ to investigate its differentiation from the neighbors. The genome size of strain SW165 ^T^ was 2,427,227 bp with 67.59 mol% G+C content. The draft genome was assembled into 33 contigs with 2,228 protein-coding sequences and 52 RNAs ( Table 3) and is visualized as Figure 3. The genomes sizes for the *Olsenella* species were comparable to one another except for *O. urininfantis* whose was only 1.75 Mbps. However, the G+C contents strain SW165 ^T^ and *O. mediterranea* were the highest but comparable to other neighboring *Olsenella* species ( Table 3). The genome of SW165 ^T^ possessed a total of 1, 230 genes with putative function and 998 genes as hypothetical proteins. Among 1,230 genes, 823 were classified as features in subsystem, following functional categories (COGs). Majority of categories included amino acid and derivatives (172 genes), carbohydrates (163 genes), and protein metabolism (132 genes) ( *Extended data:* Supplemental Table 1 ^45^).

**Table 3.** General genome characteristic of *O. lakotia* SW165 ^T^ and its neighbors.

Species	Strain	Size (Mbp)	% G+C	CDSs	rRNA	tRNA	GenBank accession number
*O. lakotia* SW165 ^T^	DSM 107283	2.43	67.6	2228	3	49	PRJNA545153
*O. profusa*	F0195	2.72	64.2	2610	3	48	GCA_000468755.1
*O. umbonata*	DSM 22620	2.35	64.9	2060	12	57	GCA_900105025.1
*O. uli*	DSM 7084	2.05	64.7	1772	3	49	GCA_000143845.1
*O. scatoligenes*	SK9K4	2.47	62.4	2110	3	47	GCA_001494635.1
*O. urininfantis*	Marseille-P3197	1.75	64.3	1558	7	48	GCA_900155635.1
*O. phocaeensis*	Marseille-P2936	2.24	66.3	2190	3	50	GCF_900120385.1
*O. mediterranea*	Marseille-P3256	2.37	67.6	2045	6	52	GCA_900119385.1

**Figure 4.**
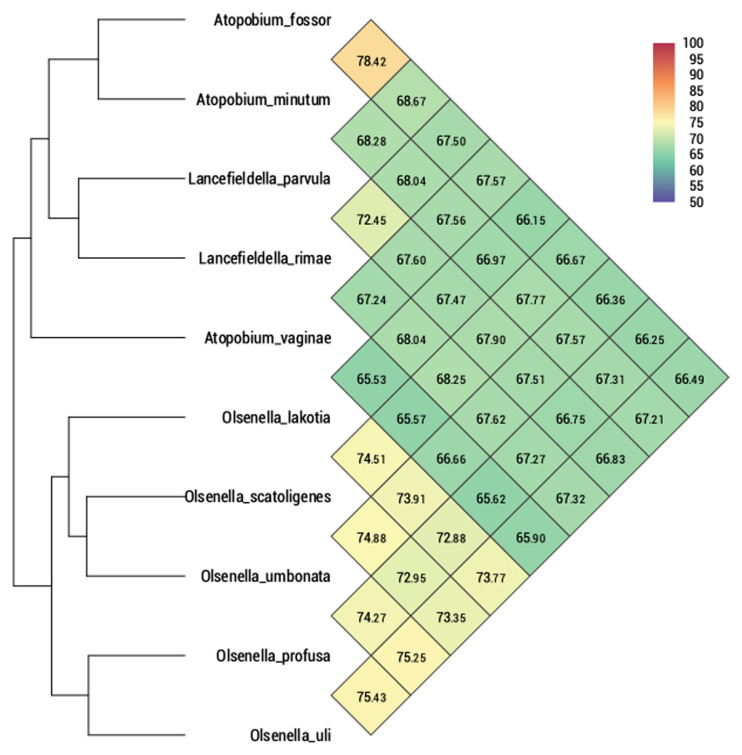
Average nucleotide Identity comparison of *O. lakotia* SW165 ^T^ and closely related strains. Heatmap represents OrthoANI values generated using OAT software between *O. lakotia* and related taxa with valid taxonomy.

**Figure 5.**
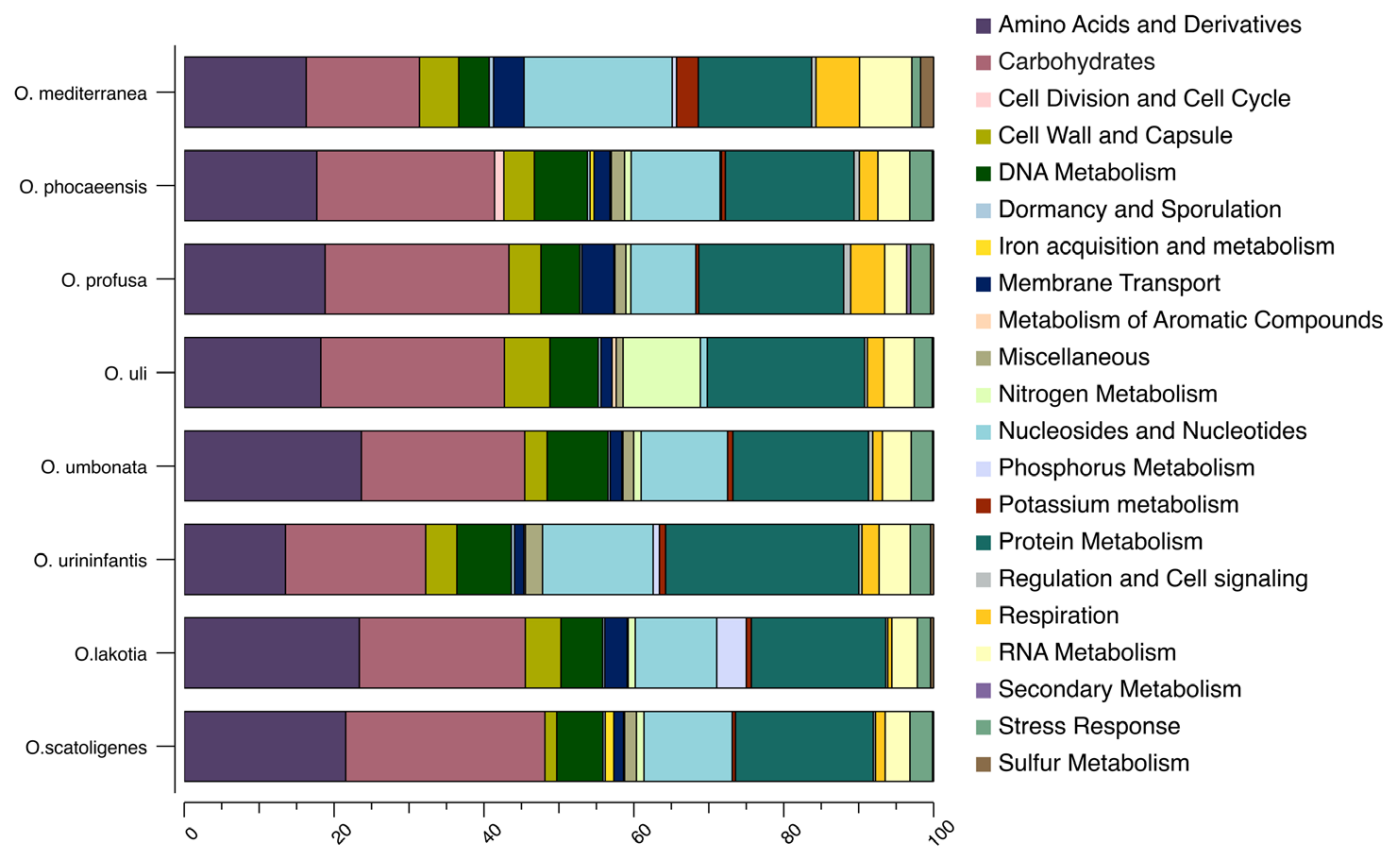
Distribution of functional features of predicted coding sequences of *O. lakotia* SW165 ^T^ and its neighbors. The functional features were predicted based on the clusters of orthologous groups. Heatmap was generated from genome annotation of individual species by RAST using Explicet software.

Furthermore, we compared the genome of SW165 ^T^ to its neighbor using OrthoANI as shown in Figure 4. The genome of SW165 was only 73.41% identical to its nearest neighbor *O. profusa* DSM 13989 ^T^. Also, the OrthoANI values for SW165 ^T^ and closely related strains ranged from 65.40 to 74.18 % ( Figure 4) indicating that the genome of SW165 ^T^ is unique compared to its neighbors. The proposed cut off for OrthoANI for the species delineation is 95-96% identity ^46, 47^. In addition, dDDH between SW165 ^T^ and the closest neighbor, *O. profusa* DSM 13989 ^T^ was only 17.6 ± 5.3. These values were lower than threshold of ANI and dDDH for delineating prokaryotic species, suggesting that these strains are distinct species. Also, the gene distribution into COGs was comparable in all eight compared *Olsenella* genomes ( Figure 5). Hence, the phenotypic and genetic discrepancy of the SW165 ^T^ with its close neighbor apparently supports that strain SW165 ^T^ represents a new species of the genus *Olsenella*.

## Discussion

Culturomics of the gut microbiota has evolved as a strong tool to increase the isolation of diverse previously uncultured bacteria from the gut ^5, 48^. The cultivation of the gut microbiota enables to improve the health through an enhanced understanding of their roles in the gut ecosystem and finally to the host. Thus, using the culturomics strategy, we were able to isolate previously uncultured bacterium SW165 ^T^ from cecal content of feral chicken and finally characterize and describe it using taxono-genomics as a novel microorganism.

The novelty of a prokaryotic organism is universally determined by the comparison of 16S rRNA gene sequence homology ^49^. The threshold values are used at distinct taxonomic levels ^46^. In this context, the newly discovered bacterium was initially validated using the full-length 16S rRNA gene sequences, which were thereafter used for taxonomic classification. Phylogenetic analysis of 16S rRNA gene showed that the novel strain SW165 ^T^ clustered with closely related taxa in the genus *Olsenella* within the family *Atopobiaceae* ( Figure 1). This genus composes of nine species, most of which are members of gut microbiota of humans and animals. However, *O. uli* is only a species that have been isolated from chicken gut ^26^. Remarkable, this study revealed a new member of *Olsenella* from gut microbiota of chicken.

Phenotypic analyses are performed to differentiate closely valid bacteria. Based on phenotypic tests, strains SW165 ^T^ appeared several distinct properties compared to other members of the genus *Olsenella* ( Table 1 and Table 2). The obvious distinct phenotypic features were observed in biochemical tests including enzymatic activity and carbon source utilization, thereby they might be important parameters for discriminating closely related species. These differences suggested the novelty of this microorganism belonging to *Olsenella*.

In addition to 16S based comparison, whole genome can be used for distinguishing, distinct bacteria. Recently, digital DNA-DNA hybridizations (dDDH) becomes a key measurement in delineation of prokaryotic species. It is an *in-silico* genome-to-genome comparison inferring whole genome distance to mimic DDH ^50^. Besides, Average Nucleotides Identification (ANI) is another particular tool that confirm the taxonomic delineation. It measures the overall similarity between two genome sequences ^51^. Recent publication of novel bacteria trend to perform genome-based analysis to support the results of 16S rRNA gene-based analysis. The strengths of genome-based analyses include comparison of all nucleotides in prokaryotic taxonomy and functional prediction ^52^. Based on genomic evidence, strain SW165 ^T^ showed low similarity in terms of OrthoANI with *Olsenella* species of the family *Atopobiaceae* ( Figure 4) *.* Further, genome features and distribution of predicted functional categories of strain SW165 ^T^ was corresponded to all other *Olsenella* species ( Figure 5 and Table 3). Thus, we proposed the strain SW165 ^T^ as a new species *Olsenella lakotia* SW165 ^T^ sp. nov., within the family *Atopobiaceae*.

### Description of Olsenella lakotia SW165 ^T^ sp. nov.

*O. lakotia* sp. nov. (la.ko’tia N.L n. referring to native American tribe). Cells are strictly anaerobic, Gram-positive streptobacillus and non-motile. The average size of each cell is 0.5–2.0 µm. Colonies are visible on BHI-M agar after 2 days and are approximately 0.2–0.5 cm in diameter, cream-white, smooth, slightly umbonate with entire circular margin. The microorganism exhibits optimal growth in BHI-M medium at 45°C and pH 7.0. The strain utilizes arbutin, cellobiose, dextrin, D-fructose, L-fucose, D-galactose, α-D-glucose, maltose, D-mannose, D-melibiose, D-raffinose, salicin, sucrose and turanose as a carbon source. Positive enzymatic reactions are obtained for alkaline phosphatase, leucine arylamidase, cysteine arylamidase, α-galactosidase, β-galactosidase, β-glucuronidase, α-glucosidase and β-glucosidase. The volatile fatty acid produced by this strain is acetic acid. The primary cellular fatty acids are C _12 : 0_, C _14 : 0_, C _14 : 0_ DMA and summed feature 1. The genome is 2,427,227 bp and its G+C is 67.59 mol%.

The type strain SW165 ^T^ (=DSM 107283 =CCOS 1887) was isolated from the cecum of feral chicken was deposited in the DSMZ and CCOS collections under accession numbers DSM 107283 and CCOS 1887 ( *Extended data*: Supplemental Data 1 ^53^), respectively. The 16S rRNA and genome sequence are available in GenBank under accession numbers MK963074 and BioProject PRJNA545153, respectively.

## Data availability

### Underlying data

*Olsenella* sp. strain SW165 16S ribosomal RNA gene, partial sequence, Accession number MK963074: https://www.ncbi.nlm.nih.gov/nuccore/MK963074

*Olsenella lakotia* SW165 Genome sequencing and assembly, Accession number PRJNA545153: https://www.ncbi.nlm.nih.gov/bioproject/PRJNA545153

### Extended data

Figshare: Extended data; Supplemental Table 1 (Functional categories (COGs) from genome of strain SW165 ^T^), https://doi.org/10.6084/m9.figshare.12793544.v1 ^45^.

Figshare: Supplemental Data 1 (DSMZ and CCOS accession numbers of strain SW165 ^T^), https://doi.org/10.6084/m9.figshare.12793610.v1 ^53^.

Data are available under the terms of the Creative Commons Zero "No rights reserved" data waiver (CC0 1.0 Public domain dedication).
