# Supplementary material for: Taxono-genomics description of Olsenella lakotia SW165 T sp. nov., a new anaerobic bacterium isolated from cecum of feral chicken
Source: F1000Res. 2020 Sep 8;9:1103. [Version 1] doi: 10.12688/f1000research.25823.1 (PMC7520715; doi:10.12688/f1000research.25823.1)
Supplement: Peer review history

# Peer review: Reviewer response for version 1

**DOI:** 10.5256/f1000research.28500.r71021

**Published:** 2020-09-24

**Authors:** Mohamed Seleem

**Affiliations:** 1 Virginia Polytechnic Institute and State University, Blacksburg, USA

This manuscript describes the isolation and characterization of a proposed new species “lakotia” within the genus Olsonella. The isolate has been characterized using the combination of genome sequencing and phenotypic assays. The phenotypic characterization includes nutrient utilization using Biolog plates, enzyme profiling using API zyme, and cellular membrane fatty acid profiling using FAME. At the phenotypic level, the proposed new species show clear differences with Olsonella umbonata, the closest taxonomic neighbor. At the genomic level, the proposed new species is only 74% similar to other species in the genus Olsonella. Therefore, the proposed species meets the genomic and phenotypic requirements of being designated as a new species. However, some minor changes could improve the clarity of the manuscript:

- Table 1. Tittle of the table reads “ Characteristics of *O. lakotia* and closely related strains” Authors should instead give the phenotype reported here. Therefore, please revise the title to improve the clarity; eg. Phenotypic properties of *O. Lakota*... Likewise, in the table, column header should be “Phenotpic properties” instead of “characteristic”. Please give species names in the column headers instead of numbers.
- Table 3. Authors have given bioproject  number instead of genbank accession number for O. lakotia SW165. The genbank accession number was anot accessible from the Bioproect page in NCBI. Please remove the bioproject number and instead give Genbank SRA accession number here.

Is the work clearly and accurately presented and does it cite the current literature?

Yes

If applicable, is the statistical analysis and its interpretation appropriate?

Not applicable

Are all the source data underlying the results available to ensure full reproducibility?

Yes

Is the study design appropriate and is the work technically sound?

Yes

Are the conclusions drawn adequately supported by the results?

Yes

Are sufficient details of methods and analysis provided to allow replication by others?

Yes

Reviewer Expertise:

Microbiology

I confirm that I have read this submission and believe that I have an appropriate level of expertise to confirm that it is of an acceptable scientific standard.

# Peer review: Reviewer response for version 1

**DOI:** 10.5256/f1000research.28500.r71020

**Published:** 2020-09-11

**Authors:** Yung-Fu Chang

**Affiliations:** 1 Department of Population Medicine and Diagnostic Sciences, College of Veterinary Medicine, Cornell University, Ithaca, NY, USA

This manuscript “ Taxono-genomics description of *Olsenella lakotia* SW165 T sp. nov., a new anaerobic bacterium isolated from the cecum of feral chicken’’ was submitted by Scaria *et al. *The authors have characterized a new bacterium strain isolated from chicken. It is a well-written manuscript and the data is very solid to support their conclusion. I have enclosed the manuscript with a minor revision which you can see here.

Is the work clearly and accurately presented and does it cite the current literature?

No

If applicable, is the statistical analysis and its interpretation appropriate?

Yes

Are all the source data underlying the results available to ensure full reproducibility?

Yes

Is the study design appropriate and is the work technically sound?

Yes

Are the conclusions drawn adequately supported by the results?

Yes

Are sufficient details of methods and analysis provided to allow replication by others?

Yes

Reviewer Expertise:

microbial pathogenesis, molecular diagnosis,  vaccine development, genomics, and proteomics.

I confirm that I have read this submission and believe that I have an appropriate level of expertise to confirm that it is of an acceptable scientific standard.

# Response

**Published:** 2020-09-14

**Authors:** Surang Chankhamhaengdecha

**Affiliations:** Faculty of Science, Mahidol University, Thailand

Thank you for reading and correcting our manuscript. We really appreciate you approval.
